# CRIF1 deficiency suppresses endothelial cell migration via upregulation of RhoGDI2

**DOI:** 10.1371/journal.pone.0256646

**Published:** 2021-08-26

**Authors:** Harsha Nagar, Seonhee Kim, Ikjun Lee, Su-Jeong Choi, Shuyu Piao, Byeong Hwa Jeon, Minho Shong, Cuk-Seong Kim

**Affiliations:** 1 Department of Physiology and Medical Science, School of Medicine, Chungnam National University, Daejeon, Republic of Korea; 2 Department of BK21 Plus CNU Integrative Biomedical Education Initiative, School of Medicine, Chungnam National University, Daejeon, Republic of Korea; 3 Research Center for Endocrine and Metabolic Diseases, School of Medicine, Chungnam National University, Daejeon, Republic of Korea; Medical College of Wisconsin, UNITED STATES

## Abstract

Rho GDP-dissociation inhibitor (RhoGDI), a downregulator of Rho family GTPases, prevents nucleotide exchange and membrane association. It is responsible for the activation of Rho GTPases, which regulate a variety of cellular processes, such as migration. Although RhoGDI2 has been identified as a tumor suppressor gene involved in cellular migration and invasion, little is known about its role in vascular endothelial cell (EC) migration. CR6-interacting factor 1 (CRIF1) is a CR6/GADD45-interacting protein with important mitochondrial functions and regulation of cell growth. We examined the expression of RhoGDI2 in CRIF1-deficient human umbilical vein endothelial cells (HUVECs) and its role in cell migration. Expression of RhoGDI2 was found to be considerably higher in CRIF1-deficient HUVECs along with suppression of cell migration. Moreover, the phosphorylation levels of Akt and CREB were decreased in CRIF1-silenced cells. The Akt-CREB signaling pathway was implicated in the changes in endothelial cell migration caused by CRIF1 downregulation. In addition to RhoGDI2, we identified another factor that promotes migration and invasion of ECs. Adrenomedullin2 (ADM2) is an autocrine/paracrine factor that regulates vascular tone and other vascular functions. Endogenous ADM2 levels were elevated in CRIF1-silenced HUVECs with no effect on cell migration. However, siRNA-mediated depletion of RhoGDI2 or exogenous ADM2 administration significantly restored cell migration via the Akt-CREB signaling pathway. In conclusion, RhoGDI2 and ADM2 play important roles in the migration of CRIF1-deficient endothelial cells.

## Introduction

Cell motility is essential for the maintenance of normal physiological and pathological processes and proper organization of multi-cellular organisms. Cell migration is fundamental during both embryonic development as well as metastasis of cancer cells. Apart from the development of multi-cellular organisms, cell migration is also important for wound healing, tissue homeostasis, and immune responses in physiological conditions, while aberrant cell migration is found in numerous pathological conditions [1]. Therefore, cell motility and migration play important roles in both normal biology and in disease. On the one hand, migration allows cells to generate complex tissues and organs during development, but on the other hand, the same mechanisms are used by tumor cells to move and spread, causing metastasis.

Rho GDP-dissociation inhibitor (RhoGDI) is a downregulator of Rho family GTPases that prevents nucleotide exchange and membrane association [2]. There are three isoforms of RhoGDI: RhoGDI1, RhoGDI2, and RhoGDI3. RhoGDI1 and RhoGDI2 are cytosolic isoforms whereas RhoGDI3 is a non-cytosolic isoform. The Rho GDP dissociation inhibitor 2 (RhoGDI2) is expressed primarily in hematopoietic and endothelial cells [3] and is differentially expressed in some human cancers [4, 5]. It contains the central regulatory molecules for the activation of Rho GTPase, which participates in the regulation of cellular processes such as migration, apoptosis, and the cell cycle [6, 7]. Although the role of RhoGDI2 in tumor cells has been established, its importance in vascular endothelial cell migration is unclear.

ECs and vasoactive molecules play key roles in the maintenance of vascular homeostasis [8]. A variety of bioactive molecules—including natriuretic peptide, nitric oxide, prostacyclin, and adrenomedullin (ADM)—are actively secreted by ECs. Originally identified as a vasodilating peptide from human pheochromocytoma [9], ADM is secreted by ECs, various tissues and organs, and is involved in a number of biological processes [10–18]. ADM promotes migration and invasion by HUVECs in a dose-dependent manner [19]. Adrenomedullin2 (ADM2) is a novel member of the calcitonin gene-related peptide (CGRP) superfamily, having 33% sequence homology with ADM [20]. ADM2 is expressed in a variety of tissues and organs, including the brain, heart, and endothelium [21]. The biological effects of ADM and ADM2 are mediated via members of the G-protein-coupled receptor family, calcitonin receptor-like receptors (CRLRs), in association with one of the receptor activity-modifying proteins (RAMP 1, 2, or 3) [20, 22].

Several signaling pathways; *e*.*g*., the Akt signaling pathway, downstream of the Rho proteins regulate cytoskeletal dynamics. Akt/protein kinase B (PKB) is a major downstream effector of growth factor receptor tyrosine kinases that signals through phosphatidylinositol-3 kinase (PI3K) [23]. The PI3K/Akt pathway plays a major role in key cellular processes, such as survival and apoptosis, cell cycle progression, glucose metabolism, and gene expression [24]. PI3K/Akt signaling induces cell proliferation and promotes their survival and motility. Furthermore, cAMP response element-binding protein (CREB) is a ubiquitously expressed nuclear transcription factor that is activated by various extracellular stimuli. CREB regulates the expression of genes important for the proliferation, differentiation, migration, and survival of several cell types [25]. Akt/PKB is reported to potently induce ser-133 phosphorylation of CREB [26].

CR6-interacting factor 1 (CRIF1) is a unique protein that is ubiquitously expressed in humans. Under normal conditions, CRIF1 localizes exclusively to the nucleus, where it colocalizes with Gadd45γ [27]. CRIF1 has distinctive functions in both the nucleus and mitochondrion [28, 29]. It also acts as a novel coregulatory protein in transactivation of the orphan nuclear receptor Nur77. In the nucleus, its primary function is to regulate cell cycle progression and cell growth [30]; moreover, it functions as a transcriptional coactivator of STAT3, influencing a variety of biological functions including transcription, DNA binding, and cellular transformation [31]. As a result, CRIF1 knockout embryos show lethality and developmental arrest, as well as defective proliferation and severe apoptosis. In the mitochondrion, CRIF1 is associated with large mitoribosomal subunits and is responsible for the production of oxidative phosphorylation (OXPHOS) polypeptides and their subsequent insertion into the inner mitochondrial membrane [32]. Therefore, both CRIF1 knockout mice and CRIF1 knockdown *in vitro* display features of mitochondrial dysfunction [33]. In the present study, we investigated the roles of RhoGDI2 and ADM2 in the migration of CRIF1-deficient HUVECs and the proteins and pathways involved in the process.

## Materials and methods

### Cell culture and transfection

Human umbilical vein endothelial cells (HUVECs) were purchased from Clonetics (San Diego, CA, USA) and cultured in Endothelial Growth Medium-2 from Lonza (Walkersville, MD, USA) according to the manufacturer’s instructions at 37°C with 5% CO_2_. Sub-confluent, proliferating HUVECs at passages 2–8 were used. HUVECs were transfected with short interfering RNA (siRNA) for CRIF1 (human siRNA sequence: sense-5’-UGGAGGCCGAAGAACGCGAAUGGUA-3’ and antisense-5’-UACCAUUCGCGUUCUUCGGCCUCCA-3’) (Cosmogenetech, Daejeon, South Korea) and negative control siRNA using Lipofectamine 2000 reagent from Invitrogen (Carlsbad, CA, USA) as per the manufacturer’s recommendations. The cells were incubated at 37°C in a 5% CO_2_ incubator for 48 h for gene knockdown.

### Antibodies, immunoblotting, and reagents

Anti-CRIF1, anti-phospho Akt, anti-phospho CREB and anti-total CREB antibodies were purchased from Santa Cruz Biotechnology (Santa Cruz, CA, USA). Anti RhoGDI2 antibody was purchased from Spring Bioscience (Pleasanton, California, USA). Anti-β-actin and anti-total Akt antibodies were from Cell Signaling Technology (Beverly, MA, USA). Western blot analysis was performed by adding 30 μg of cell lysate to sodium dodecyl sulfate-polyacrylamide gel electrophoresis loading buffer followed by boiling and separation by electrophoresis and transfer onto nitrocellulose membranes. After incubation with appropriate primary and peroxidase-conjugated secondary antibodies (Santa Cruz Biotechnology), the chemiluminescent signal was developed using Super Signal West Pico or Femto Substrate (Thermo Fisher Scientific, Pierce, Rockford, IL). Blots were imaged and band densities quantified with a Gel Doc 2000 Chemi Doc system using Quantity One software (Bio-Rad, Hercules, CA). Values were normalized relative to β-actin as the loading control. Recombinant ADM2 (Intermedin-53, human) was purchased from Phoenix Pharmaceuticals Inc. (Burlingame, CA, USA).

### Wound healing assay

A wound healing assay was used to assess cell migration. Cells were transfected as described previously with negative control or CRIF1 siRNA in six-well tissue culture plates for 24 h, after which a sterile 200 μL pipette tip was used to detach the cells from the monolayer across the center of the well. Floating cells were flushed out by gently rinsing twice with PBS and replaced with serum-free medium (to rule out cell proliferation as the cause of wound closure) followed by incubation for another 24 h. The total incubation time post transfection was therefore 48 h. Cell movement was monitored using microscopy. Photographs were taken immediately and at 24 h after scratching. The relative wound area was quantitatively evaluated using ImageJ software (NIH, Bethesda, MD, USA).

### Transwell assay

Transwell assay was employed to detect cell migration. Cells were transfected with negative control or CRIF1 siRNA in 6-well tissue culture plates for 24 h, followed by transfer of 5 X 10^5^/ml cells in the upper transwell chamber (24-well plate; Corning, New York, USA) and culture with FBS free medium. Complete growth medium with 10% FBS was added to the lower chamber and incubated for another 24 h. Then, cells on the upper side (nonmigrating cells) were removed and migrated cells on the lower face were washed with PBS, fixed with 4% paraformaldehyde, stained with DAPI and counted on 5 random high-power fields (200x magnification) under a microscope and averaged.

### Real-time polymerase chain reaction

Total RNA was isolated from cells using TRIzol reagent (Thermo Fisher Scientific). Complementary DNA (cDNA) was generated using an RT premix kit (iNtRON Biotechnology, Seongnam, Republic of Korea). Relative RNA levels were determined by PCR using a SYBR qPCR premix (Enzynomics, Daejeon, Republic of Korea). The primer sequences were as follows: GAPDH: 5’-GGAGCGAGATCCCTCCAAAAT-3’ (forward) and 5’-GGCTGTTGTCATACTTCTCATGG-3’ (reverse); ADM2: 5’-GCCAGGTGCAGAATCTCAG-3’ (forward) and 5’-ATAGCTGTGGGGGCTGCT -3’ (reverse); RAMP2: 5’-CGACTGGGCCATGATTAGCA-3’ (forward) and 5’-AGGAGTACATCCTCTGGGGG-3’ (reverse); RAMP3: 5’-CTCTGCGGTGGGTGTCC -5’ (forward) and reverse-5’-TCCACCTTGCCCATCATGTC-3’; and CRIF1: 5’-GCACGCAGCCTACTAGGTG-3’ (forward) and 5’-CGAACTGCTTAGCCGCGTA-3’ (reverse). The PCR cycling conditions were as follows: 5 min at 95°C, 40 cycles of 30 s at 95°C, 30 s at 60°C and 30 s at 72°C, followed by 5 min at 72°C. GAPDH was used as an internal control. Results were interpreted by the relative quantity method (ΔΔCt).

### Statistical analysis

Statistical analysis was performed using SPSS 24.0 statistical software (Chicago, IL, USA), and graphs were generated using GraphPad Prism 6 software (GraphPad Software, La Jolla, CA, USA). Differences between groups were evaluated using two-tailed Student *t*-tests (for binary groups). For multiple comparisons, one-way analysis of variance (ANOVA) was performed followed by Tukey’s *post hoc* test. Data are presented as means ± standard error of the mean. P ≤ 0.05 was considered indicative of statistical significance. Data are representative of at least three independent experiments.

## Results

### CRIF1 knockdown inhibits cell migration and enhances RhoGDI2 expression in HUVECs

**Fig 1:**
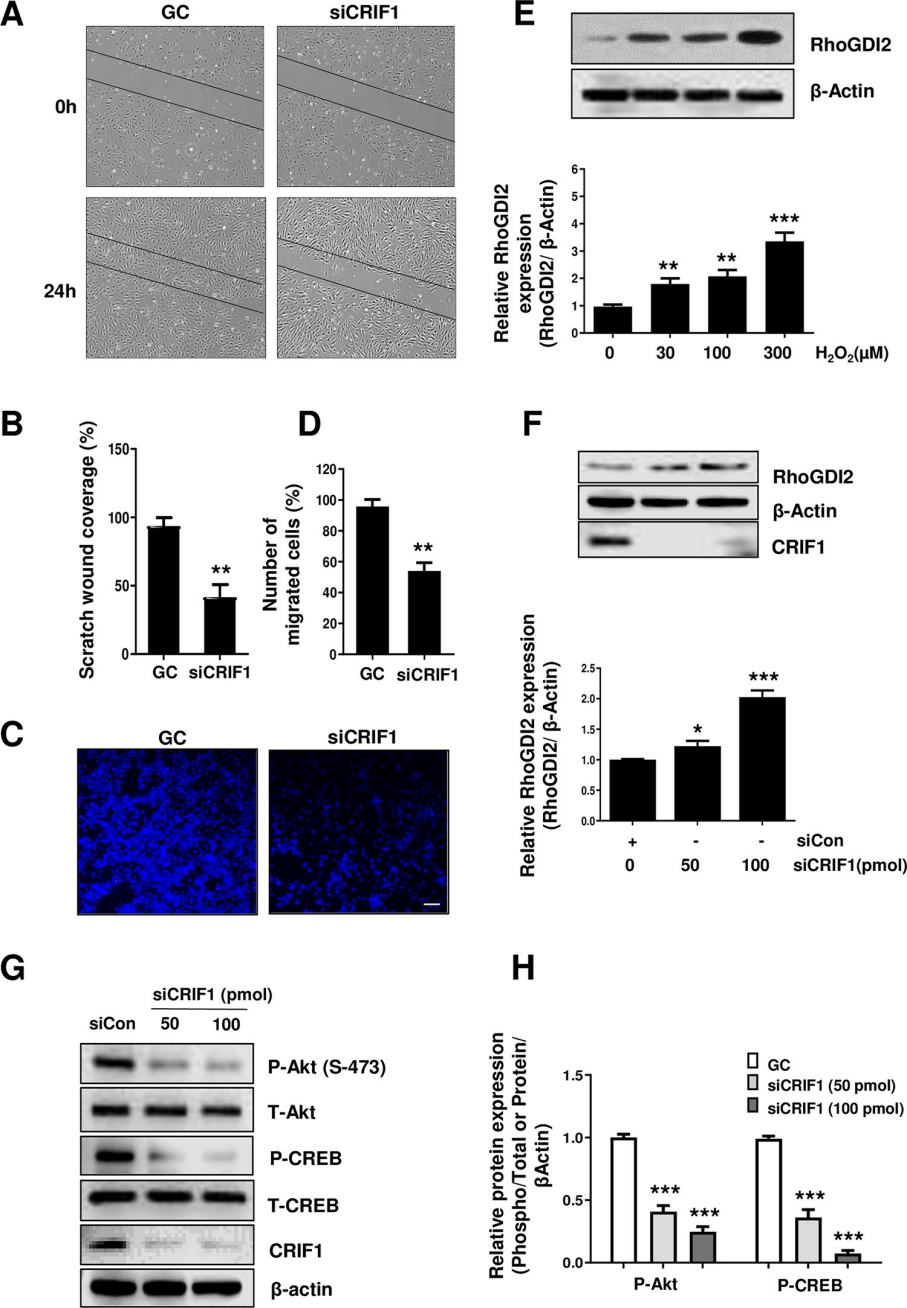
Effect of CRIF1 deficiency on cell migration and RhoGDI2 expression in endothelial cells. (A) HUVECs were cultured in six-well plates, transfected with control or CRIF1 siRNA (100 pmol), and incubated for 24 h. Next, the cells were wounded for 24 h. Images were obtained using a light microscope. (B) Quantification of wound closure was performed using ImageJ software. (C) HUVECs were transfected with control or CRIF1 siRNA (100 pmol) and transwell assay was conducted to determine cell migration. Scale bar 200 μm. (D) Quantification of the number of migrated cells was performed using ImageJ software. (E) Western blot analysis of RhoGDI2 in HUVECs treated with the indicated doses of H_2_O_2_ for 24 h. β-actin was used as an internal control. RhoGDI2 protein level quantified by densitometric analysis using ImageJ software is shown in the down panel. (F) Western blot analysis of RhoGDI2 after 48 h of dose-dependent CRIF1 siRNA transfection. β-actin was used as an internal control. RhoGDI2 protein level quantified by densitometric analysis using ImageJ software is shown in the down panel. (G) Western blot of phospho-Akt and phospho-CREB levels in HUVECs after 48 h of CRIF1 siRNA transfection. β-actin was used as an internal control. (H) Protein levels were quantified by densitometric analysis using ImageJ software. Data are means ± SD of three independent experiments. *P < 0.05, **P < 0.01 and *** *P* < 0.001 relative to the control (n = 3 per group).

To determine cell migration in CRIF1-silenced HUVECs, a wound healing assay was performed using control and CRIF1 siRNA-treated HUVECs. Cells were seeded in six-well plates, transfected with control or CRIF1 siRNA, and incubated for 24 h, after which a scratch was made through the center of each well. Photographs were taken at baseline and at 24 h after scratching (Fig 1A and 1B). CRIF1 knockdown resulted in a marked decrease in spontaneous wound healing. The wound area in control siRNA-treated cells was almost covered by migrating cells by 24 h, but an open area remained in the CRIF1 siRNA-treated cells.

We also performed transwell assay to detect cell migration. Cells transfected with control or CRIF1 siRNA were incubated for 24 h, after which they were transferred to the upper transwell chamber in serum free medium. The lower chamber contained complete medium. After 24 h of incubation, the migrated cells were stained with DAPI and counted under a microscope. As shown in Fig 1C and 1D, it was confirmed that cell migration was reduced by CRIF1 knockdown.

Rho GTPases are key regulators of cell adhesion and migration. They remodel cytoskeletal components, thus influencing cell mobility [34]. RhoGDI2 is reportedly a metastasis suppressor gene and a prognostic indicator in bladder cancer [35]. It has been shown previously that elevated levels of RhoGDI2 inhibit the migration of astrocytes [36]. As well as protein regulatory factors, small molecule redox agents such as superoxide and H_2_O_2_ (ROS) regulate the activity of redox-active RhoGTPases [37–39]. To demonstrate the involvement of ROS in the activation of RhoGDI2, we treated HUVECs with 30 to 300 μM H_2_O_2_ for 24 h and assayed RhoGDI2 protein levels using western blotting. H_2_O_2_ dose-dependently increased RhoGDI2 protein levels (Fig 1E). We have previously shown that CRIF1 knockdown leads to increase in ROS levels in HUVECs [33]. Next, we used siRNA for the knockdown of CRIF1 in HUVECs, and assayed RhoGDI2 protein levels. As expected, RhoGDI2 protein levels were increased dose-dependently by CRIF1 knockdown (Fig 1F). Therefore, RhoGDI2 is implicated in the regulation of migration of CRIF1-silenced HUVECs.

### CRIF1 knockdown inhibits the Akt-CREB signaling pathway in CRIF1 silenced HUVECs

As the end point of the PI3K pathway, Akt activation contributes to the malignant transformation of several cell types [40]. The PI3K/Akt pathway has cross talk with RhoGDI2 in lung cancer metastasis [41]. To explain the relationship between RhoGDI2 and the PI3K/Akt pathway in CRIF1-silenced HUVECs, we assayed phosphorylated Akt levels in CRIF1—silenced cells. Ser-473 phosphorylation level of Akt was decreased dose-dependently by siRNA-mediated knockdown of CRIF1 (Fig 1G and 1H). Next, activation of CREB requires phosphorylation of serine residue 133, which is induced by a range of stimuli via distinct signaling pathways [42]. Similar to the decrease in Akt phosphorylation levels, ser-133 phosphorylation level of CREB was dose-dependently decreased by CRIF1 downregulation (Fig 1G and 1H).

### Effect of RhoGDI2 knockdown on the activation of Akt-CREB signaling pathway and migration in CRIF1 silenced HUVECs

**Fig 2:**
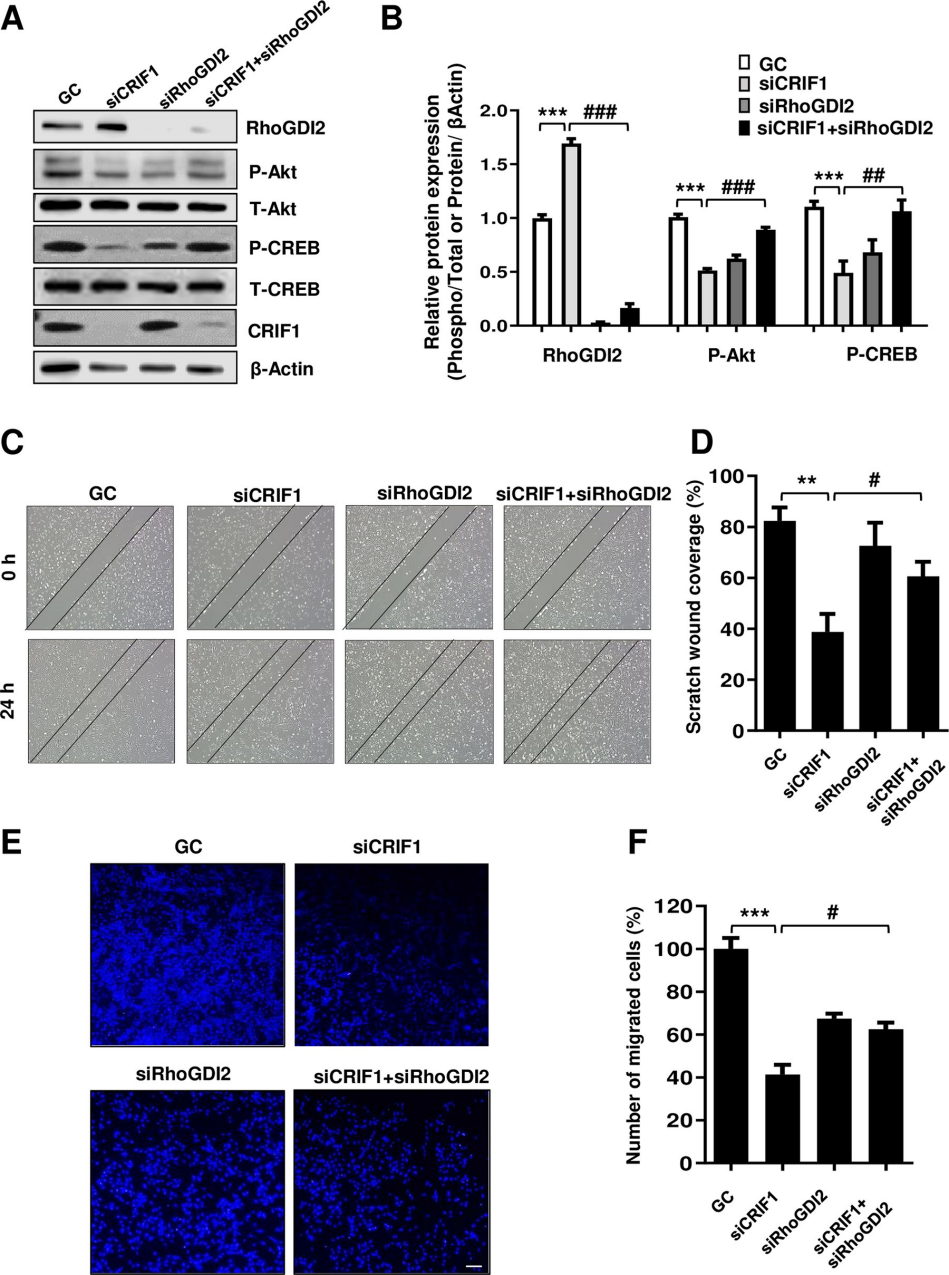
RhoGDI2 knockdown in CRIF1-silenced HUVECs leads to activation of the Akt-CREB signaling pathway and restoration of endothelial cell migration. (A) HUVECs were transfected with the control, CRIF1 siRNA, or co-transfected with CRIF1 and RhoGDI2 siRNA (100 pmol) for 48 h followed by western blot analysis of phospho-Akt and phospho-CREB levels. (B) Protein levels were quantified by densitometric analysis using ImageJ software. (C) HUVECs were transfected with the control, CRIF1 siRNA, or co-transfected with CRIF1 and RhoGDI2 siRNA (100 pmol) and incubated for 24 h. Next, the cells were wounded for 24 h. Images were obtained using a light microscope. (D) Quantification of wound closure was performed using ImageJ software. (E) HUVECs were transfected with the control, CRIF1 siRNA, or co-transfected with CRIF1 and RhoGDI2 siRNA (100 pmol) and transwell assay was conducted to determine cell migration. Scale bar 200 μm. (F) Quantification of the number of migrated cells was performed using ImageJ software. Data are means ± SD of three independent experiments. **P < 0.01 and *** *P* < 0.001 relative to the control, #*P* < 0.05, ##*P* < 0.01 and ### *P* < 0.001 relative to siCRIF1 (n = 3 per group).

To elucidate the role of RhoGDI2 in cell migration and the involvement of Akt-CREB signaling pathway in the process, we used a siRNA to suppress RhoGDI2 expression. Knockdown of RhoGDI2 by the siRNA increased Akt and CREB phosphorylation levels in CRIF1-silenced HUVECs (Fig 2A and 2B). Moreover, cell migration was also restored in CRIF1 and RhoGDI2 double knockdown condition (Fig 2C–2F). Therefore, knockdown of RhoGDI2 increased the migration of CRIF1-silenced HUVECs by activating the Akt-CREB signaling pathway.

### CRIF1 knockdown increases the mRNA levels of RAMPs and endogenous ADM2 in HUVECs

**Fig 3:**
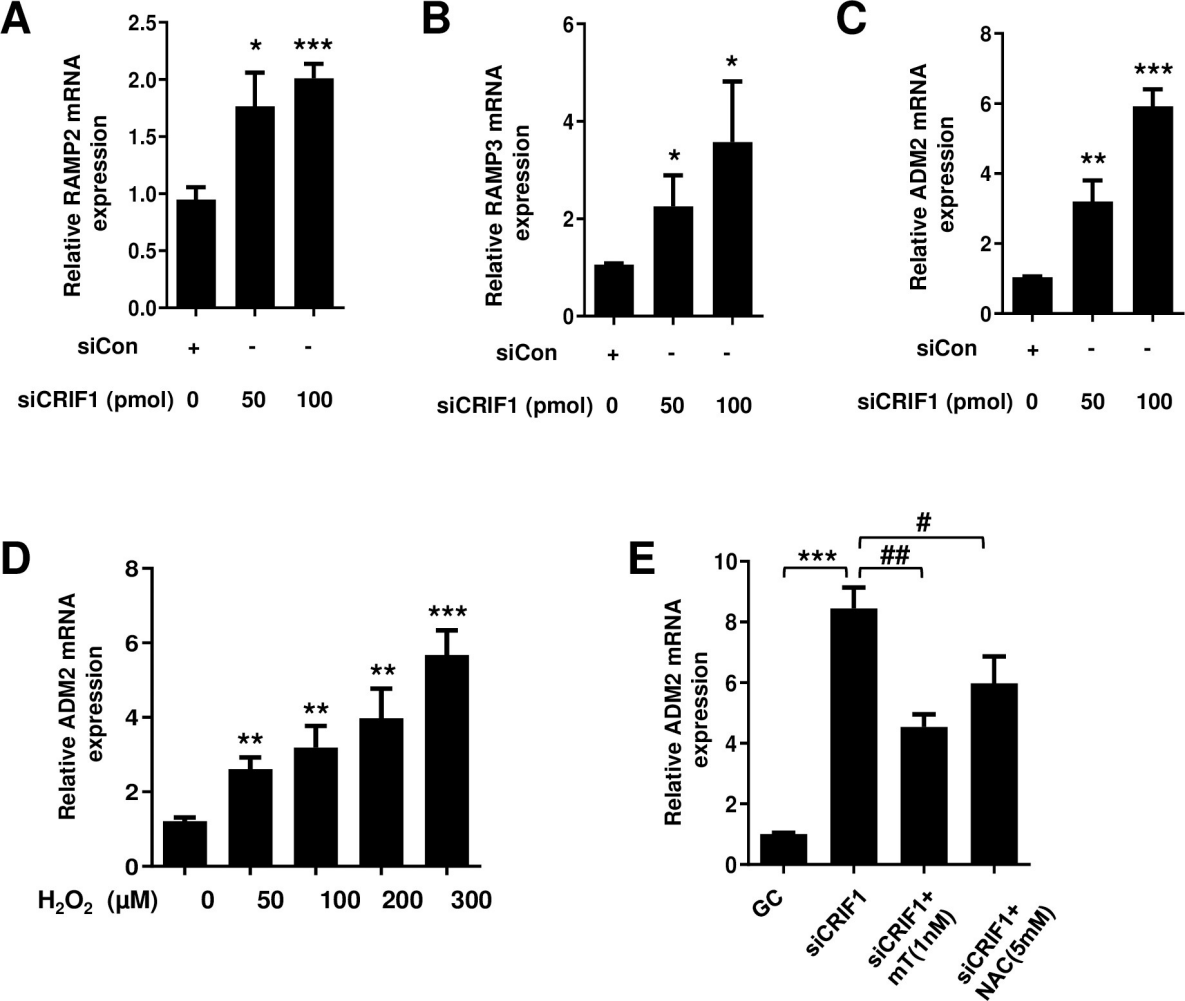
Effect of CRIF1 deficiency on the mRNA levels of RAMPs and endogenous ADM2 in endothelial cells. HUVECs were transfected with CRIF1 siRNA for 48 h. Next, the mRNA levels of (A) RAMP2 (B) RAMP3 and (C) ADM2 were determined by qPCR. (D) HUVECs were treated with the indicated doses of H_2_O_2_ for 24 h followed by the detection of ADM2 mRNA levels (E) HUVECs were pretreated with the antioxidants MitoTEMPO (1 nM) and NAC (5 mM) for 1 h followed by transfection with control or CRIF1 siRNA (100 pmol) for 48 h. mRNA levels of ADM2 were determined by qPCR. Data are means ± SD of three independent experiments. *P < 0.05, **P < 0.01 and *** *P* < 0.001 relative to the control, #*P* < 0.05 and ##*P* < 0.01 relative to siCRIF1 (n = 3 per group).

The biological effects of ADM and ADM2 are mediated via calcitonin receptor-like receptors (CRLRs; G protein-coupled receptors) in association with one of the receptor activity-modifying proteins (RAMP1, 2, or 3) [20, 22], with specificity for ADM2 being conferred by RAMP2 and RAMP3 [43]. ADM2 and its receptors, especially RAMP2 and RAMP3, are actively expressed in HUVECs [19]. In addition, expression of ADM2 and its receptors is increased in the heart, plasma, and cardiovascular tissue in pathological conditions such as hypertension, atherosclerosis, or congestive heart failure [44–46]. RAMP2, RAMP3 and ADM2 mRNA levels increased dose-dependently in CRIF1-silenced HUVECs (Fig 3A–3C), indicating an autocrine/paracrine role for endogenous ADM2. The binding of ADM2 to its membrane receptor triggers a cascade of biological events.

### CRIF1-knockdown-mediated increase in ADM2 expression is linked to oxidative stress in HUVECs

In the vasculature, oxidative stress is implicated in the pathophysiology of hypertension and atherosclerosis. It also induces profound dysfunction of vascular ECs. ECs regulate vascular tone and remodeling by modulating the secretion of vasoactive substances. These vasoactive substances regulate not only vascular tone but also vascular growth. ADM is one such vasoactive peptide secreted from vascular ECs [47]. To elucidate the significance of oxidative stress in the modulation of ADM2 expression, we examined the effect of H_2_O_2_ on the mRNA levels of ADM2. We treated the cells with 50–300 μM H_2_O_2_ for 24 h and assayed ADM2 mRNA levels using qPCR. H_2_O_2_ dose-dependently increased ADM2 mRNA levels in HUVECs (Fig 3D), confirming that oxidative stress triggers ADM2 activation. Next, we treated CRIF1-silenced HUVECs with the antioxidants NAC and mitoTEMPO and measured ADM2 mRNA levels. The increased ADM2 mRNA levels were reduced by antioxidant treatment (Fig 3E), confirming the involvement of oxidative stress in ADM2 signaling.

### Exogenous ADM2 enhances the mRNA levels of RAMPs and ADM2 and restores CRIF1 knockdown-induced suppression of the Akt-CREB pathway in HUVECs

**Fig 4:**
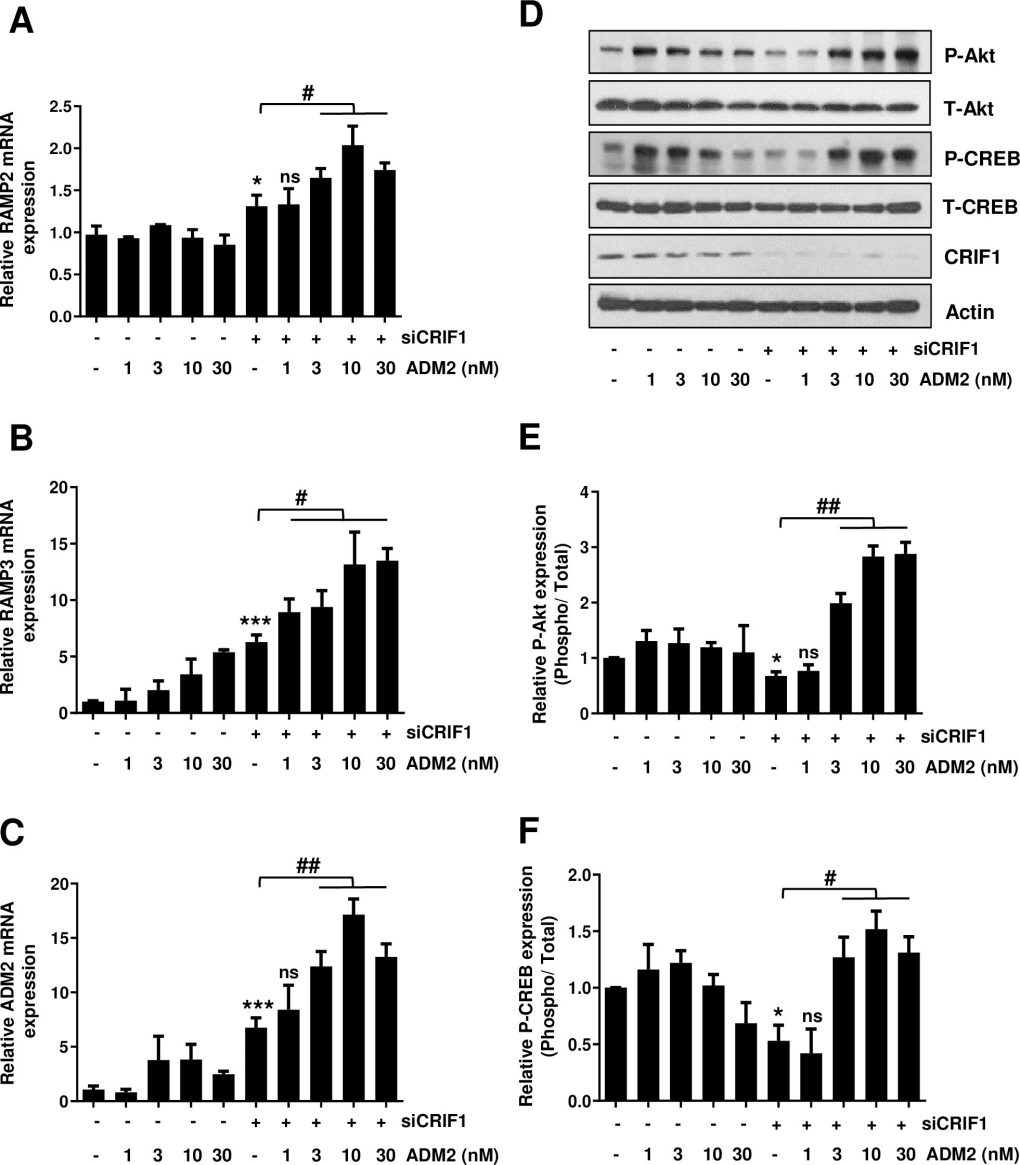
Effect of exogenous ADM2 on the Akt-CREB signaling pathway in CRIF1-silenced endothelial cells. HUVECs were transfected with control or CRIF1 siRNA (100 pmol) followed by treatment with ADM2 for 48 h. Next, the mRNA levels of (A) RAMP2 (B) RAMP3 and (C) ADM2 were determined by qPCR. (D) Western blot analysis of phospho-Akt and phospho-CREB levels. (E and F) Protein levels were quantified by densitometric analysis using ImageJ software. Data are means ± SD of three independent experiments. *P < 0.05 and *** *P* < 0.001 relative to the control, #*P* < 0.05, ##*P* < 0.01 and ns = not significant relative to siCRIF1 (n = 3 per group).

We next investigated the influence of exogenous ADM2 on RAMP and ADM2 mRNA levels. We first transfected the cells with CRIF1 siRNA and then treated 1–30 nM of ADM2 for 48 h. Exogenous ADM2 resulted in a significant increase in the RAMP2, RAMP3, and ADM2 mRNA levels in a dose-dependent manner (Fig 4A–4C).

ADM-induced Akt activation has been reported in rat aortic tissue [48] and in cultured endothelial cells [49]. We treated 1–30 nM exogenous ADM2 in CRIF1 silenced cells and measured the phosphorylation levels of Akt and CREB. Exogenous ADM2 resulted in a significant increase in phosphorylation levels of Akt and CREB in CRIF1-silenced HUVECs (Fig 4D–4F). The Akt-CREB signaling pathway regulates endothelial cell migration and survival [50]; therefore, Akt-CREB activation by exogenous ADM2 can be regarded as a key event in the transduction of angiogenic signals [51].

### Exogenous ADM2 recovers CRIF1 knockdown-induced delay in migration of HUVECs

**Fig 5:**
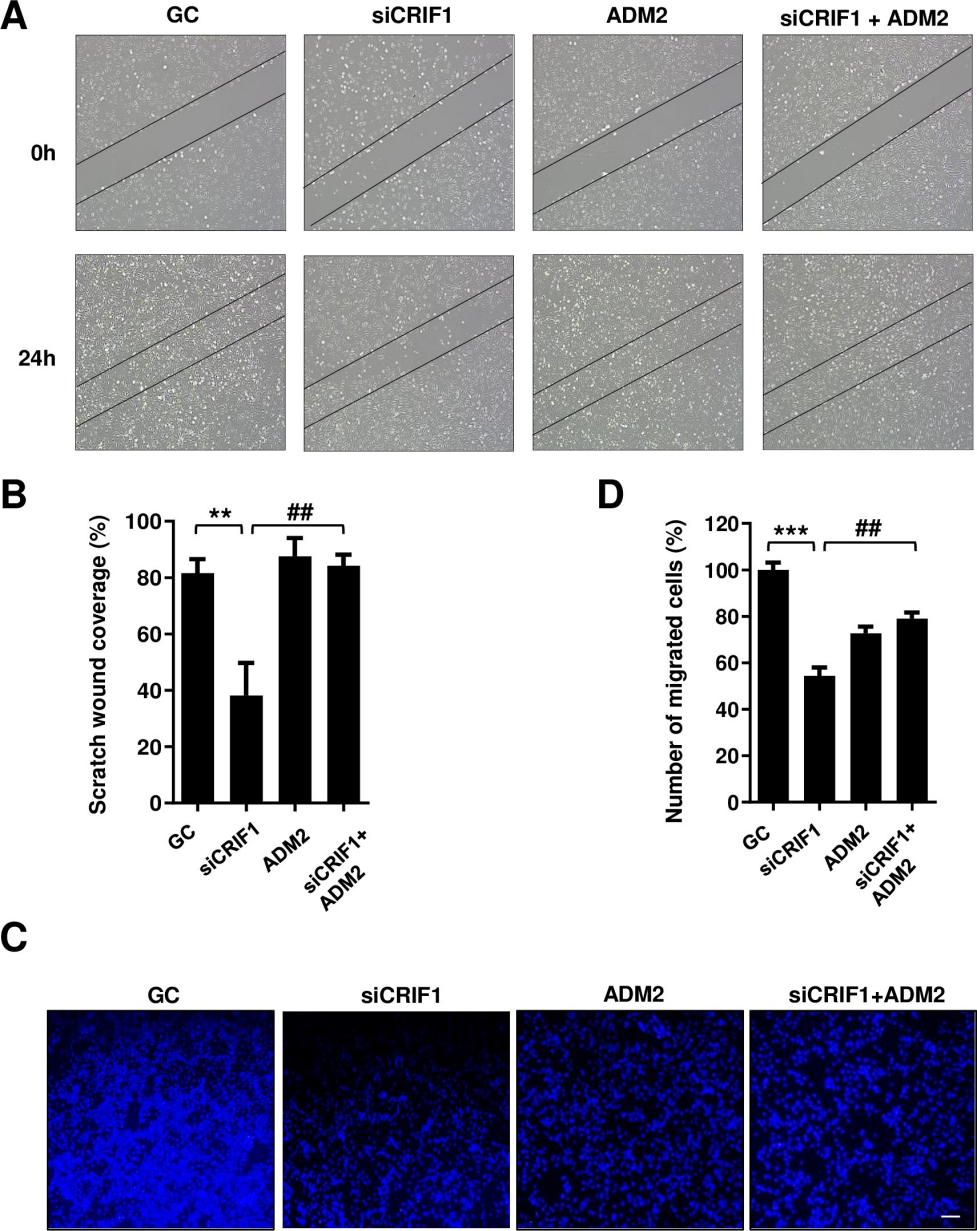
Effect of exogenous ADM2 on the migration of CRIF1-silenced endothelial cells. (A) HUVECs were transfected with control or CRIF1 siRNA (100 pmol) and treated with ADM2 for 24 h. Next, the cells were wounded for another 24 h. Images were obtained using a light microscope. (B) Quantification of wound closure was performed using ImageJ software. (C) HUVECs were transfected with control or CRIF1 siRNA (100 pmol), treated with ADM2 for 24 h.and transwell assay was conducted to determine cell migration. Scale bar 200 μm. (D) Quantification of the number of migrated cells was performed using ImageJ software. Data are means ± SD of three independent experiments. **P < 0.01 and *** *P* < 0.001 relative to the control, ##*P* < 0.01 relative to siCRIF1 (n = 3 per group).

To evaluate the effect of exogenous ADM2 on endothelial cell migration, we treated HUVECs with 10 nM exogenous ADM2 after control or CRIF1 siRNA transfection and performed a scratch wound healing assay. Wound closure was accelerated by exogenous ADM2 as compared to the control (Fig 5A and 5B). At the same time, transwell migration assay also showed increased cell migration by exogenous ADM2 administration (Fig 5C and 5D). Taken together, these results imply that exogenous ADM2 exerts a beneficial effect on the cell migration of CRIF1 silenced HUVECs.

## Discussion

**Fig 6:**
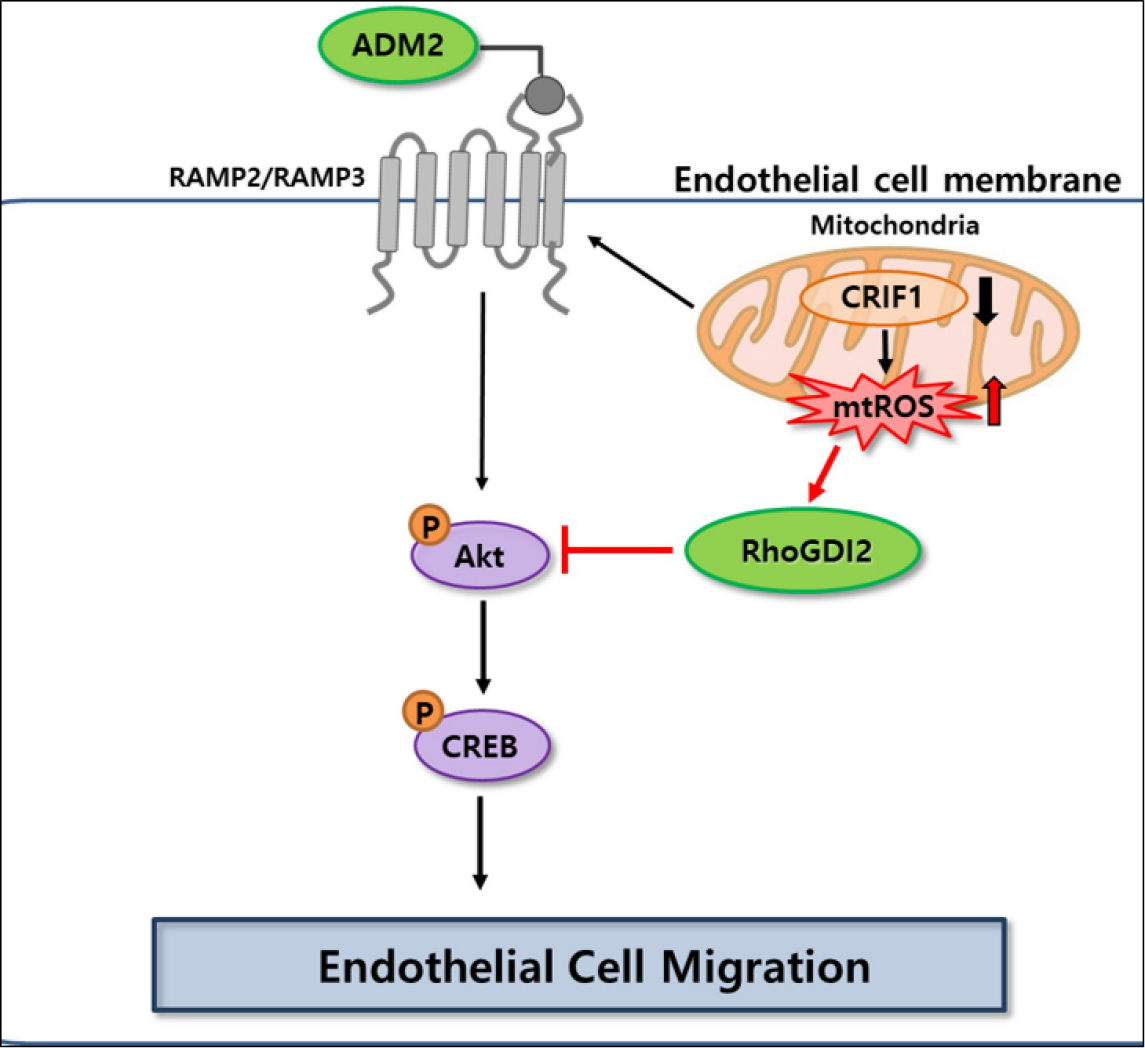
Schematic model of the pathway involved in CRIF1 knockdown induced decrease in cell migration and the possible roles of RhoGDI2 and ADM2 in endothelial cell migration.

Rho GTPase proteins influence cell mobility, as they are involved in cytoskeletal remodeling. The biological activities of Rho GTPases are governed by a tightly regulated GDP/GTP cycle, which is stimulated by guanine-nucleotide-exchange factors (GEFs) and terminated by GTPase-activating proteins. Regulation of Rho-family proteins therefore has a marked effect on metastasis, which involves cancer cell migration. In addition, RhoGDIs mediate a further level of regulation of RhoGTPases [52]. Aberrant expression of RhoGDI2 has been found in a variety of human cancers. RhoGDI2 plays dual roles in regulating the Rac GTPases activities that contribute to aggressive phenotypes of cancer cells [53]. Although RhoGDI2 regulates metastasis of several cancer types, its role and mechanism in endothelial cell migration is still unclear. In this study, RhoGDI2 expression was upregulated in CRIF1 deficient endothelial cells, which delayed cell migration via the Akt-CREB signaling pathway (Fig 6). We found that siRNA-mediated knockdown of RhoGDI2 in CRIF1-silenced endothelial cells activated the PI3K/Akt pathway by increasing the phosphorylation of Akt. Similar RhoGDI2 characteristics have been described in bladder cancer, ovarian carcinoma, and Hodgkin lymphoma [54–56]. Therefore, RhoGDI2 knockdown increases the migration of HUVECs *in vitro* because a key function of RhoGTPases is cytoskeletal remodeling, thus influencing cell motility.

ADM and ADM2 were independently discovered and a subsequent primary structural analysis showed them to be identical. They are cardiovascular polypeptides and brain-gut peptides of the CGRP superfamily, and composed of 47 amino acids [20]. ADM is an essential cardio-renal protective factor [57]. As a new member of the CGRP family, ADM2 has cardiovascular regulatory effects similar to or greater than those of ADM. As described previously, biological effects of ADM and ADM2 are mediated via CRLRs along with one of the RAMPS. RAMPs are extensively distributed in various tissues. RAMP1 is expressed at high levels in skeletal muscle, brain, and pancreas and at low levels in the liver and kidneys. RAMP2 shows high abundance in the heart and lung and low abundance in the liver and kidneys. RAMP3 is expressed at high levels in humans and low levels in rats and mice [58]. In this study, CRIF1 knockdown led to upregulation of RAMP2, RAMP3, and ADM2 mRNA levels in HUVECs. We consider that this represents a mechanism by which ADM2 compensates for CRIF1 deficiency. Indeed, it has been reported that expression of ADM2 and its receptors in the heart increases under pathological conditions such as congestive heart failure and hypertension [44, 46].

Next, we demonstrated that exogenous ADM2 treatment of CRIF1-silenced cells enhanced the mRNA levels of the receptors RAMP2 and RAMP3 as well as ADM2. Exogenous administration of ADM and CGRP peptides or their gene delivery is a new preventive and therapeutic strategy for cardiovascular diseases such as hypertension, myocardial ischemia, heart failure, and renal failure [11, 59, 60]. The therapeutic value of exogenous ADM2 depends on the formulation approaches to make sure of their activity, integrity and also precise dosing. Several drug delivery systems like polymer micro and nanoparticles have been used as carriers for the delivery of peptides or growth factors. Besides providing controlled-release properties, encapsulation of the peptides or growth factors into poly lactic-co-glycolic acid (PLGA) nanoparticles presents a suitable method for protecting the protein from surrounding microenvironment [61]. It has been shown previously that ADM induced Akt activation in rat aortic tissues and cultured endothelial cells [48, 49]. We confirmed that exogenous ADM2 treatment induces Akt and CREB activation in CRIF1-silenced HUVECs. ADM2 is involved in physiological and pathological angiogenesis in some cell lines and tissues. It induces the growth of human endometrial microvascular endothelial cells and regulates angiogenesis in the female reproductive tract [62]. Under normal conditions, there should be a balance between angiogenic stimulators and inhibitors. If this balance is broken, vascular system will be activated and angiogenesis will be excessive or degenerated. Consequently, related diseases will appear. As cell migration is a fundamental first step in angiogenesis, changes in cell migration will ultimately lead to pathologic processes such as inflammation, cardiovascular diseases, tumor development and so on. Previous studies have demonstrated the importance of cell migration to the angiogenesis process in relation to various physiological as well as pathological processes like wound healing, cancer growth and metastasis [63–65]. These studies have explained the temporal relationship between cell migration and angiogenesis and state that cell migration is a significant event associated with angiogenesis. However, the importance of endothelial cell migration and consequent angiogenesis in CRIF1 knockdown condition has not been reported. Given the significance of HUVEC migration in angiogenesis, the fact that CRIF1 knockdown induced decrease in cell migration would result in a decrease in angiogenesis cannot be ignored. In this study, CRIF1 knockdown decreased endothelial cell migration, which could be beneficial in pathophysiological conditions where there is excessive angiogenesis. Therefore, CRIF1 knockdown could be used as a therapeutic strategy in such diseases.

The limitation of this study is the use of an in vitro model for investigating the effects of CRIF1 knockdown on endothelial cell migration. For better understanding of any disease process, studying its etiology as well as progression in the host organism is an optimum method. Therefore, in vivo assays using endothelial dysfunction animal models may represent practical utilization of the findings of this study. However, the focus of this study was endothelial cell migration and it is difficult to directly demonstrate cell migration in animal models. Considering that cell migration is followed by angiogenesis, an indirect approach to demonstrate cell migration would be to study angiogenesis in vivo. Various assays could be carried out in vivo such as the matrigel assay, implantation of sponges and polymers, chamber assay or directed in vivo angiogenesis assay (DIVAA) in the future studies. Still, we have to accept the fact that these methodologies too have some limitations, which may or may not be mitigated with specific modifications. Finally, it can be concluded that CRIF1 knockdown decreased cell migration by upregulating RhoGDI2 expression. Under these conditions, RhoGDI2 knockdown or exogenous ADM2 treatment restored the migratory ability of endothelial cells.

## Supporting information

S1 Raw images (PDF)
