# Supplementary figures and images for: CRIF1 deficiency suppresses endothelial cell migration via upregulation of RhoGDI2
Source: PLoS One. 2021 Aug 26;16(8):e0256646. doi: 10.1371/journal.pone.0256646 (PMC8389428; doi:10.1371/journal.pone.0256646)

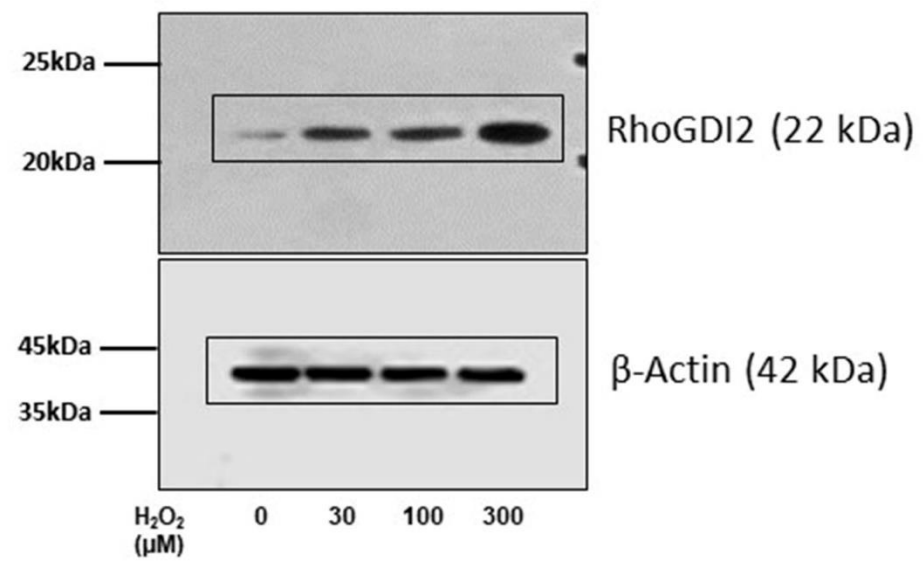

Fig 1E

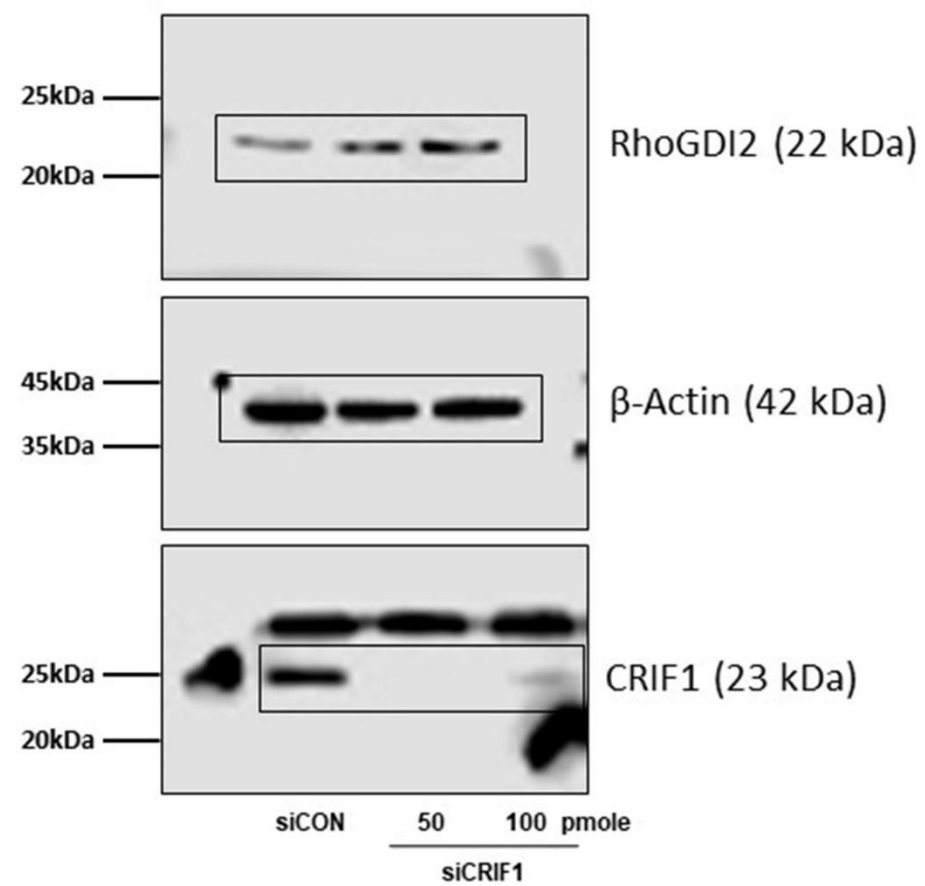

Fig 1F

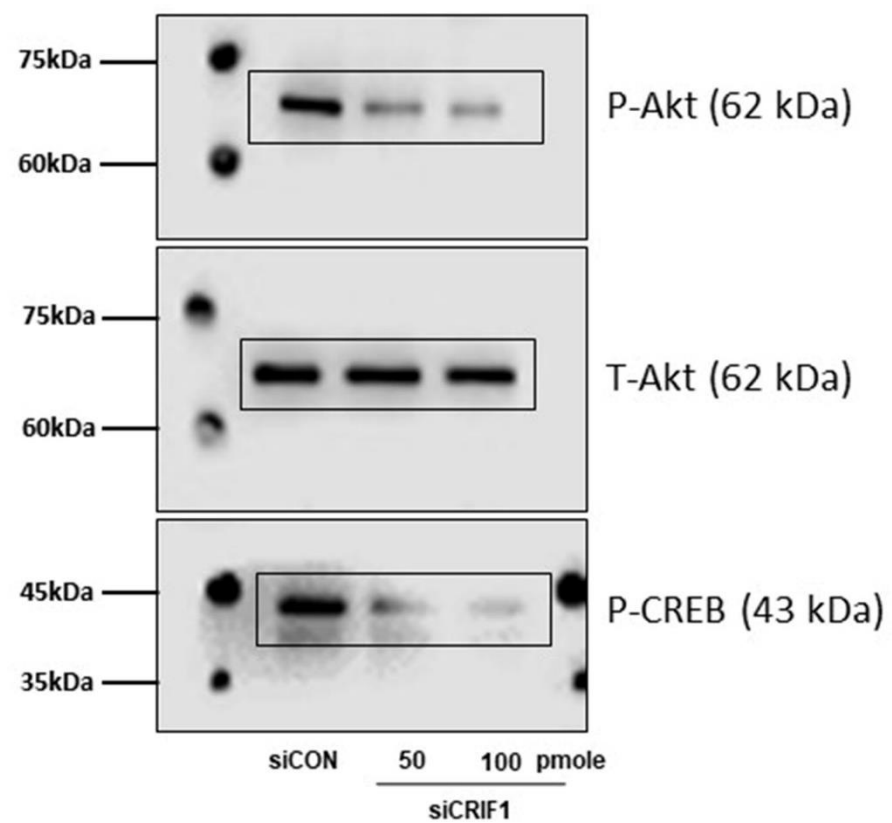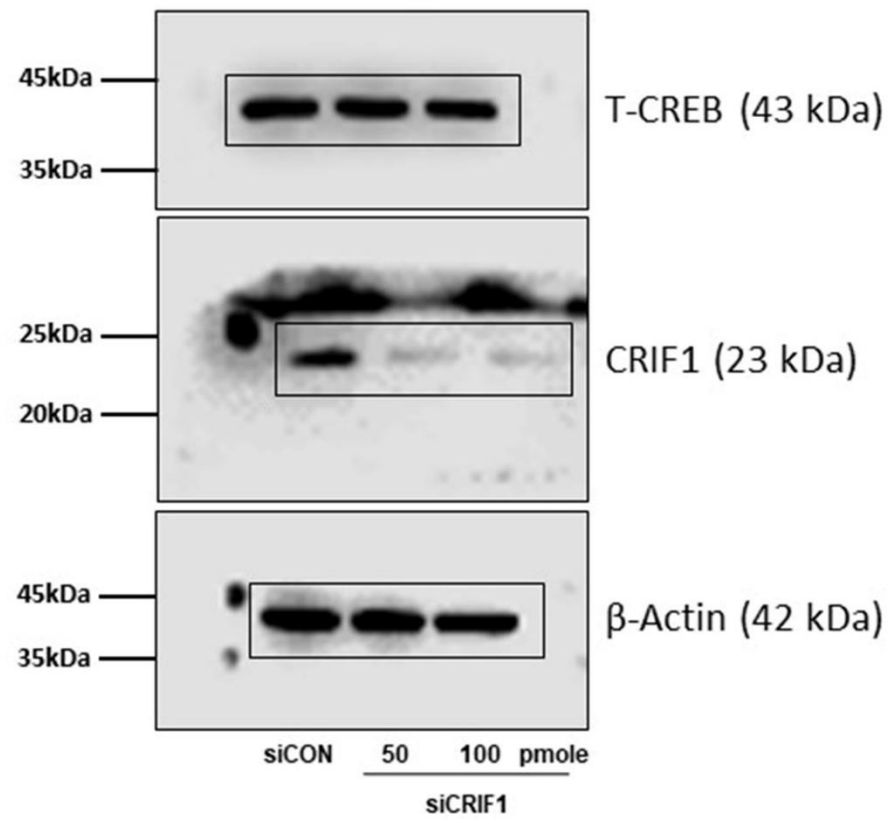

Fig 1G

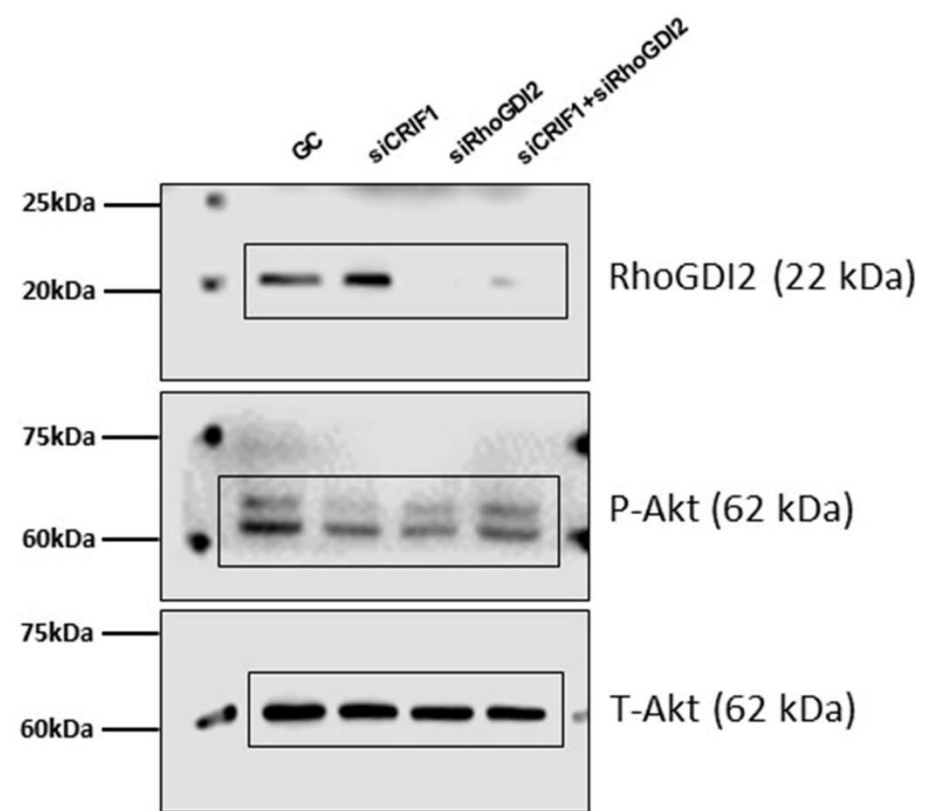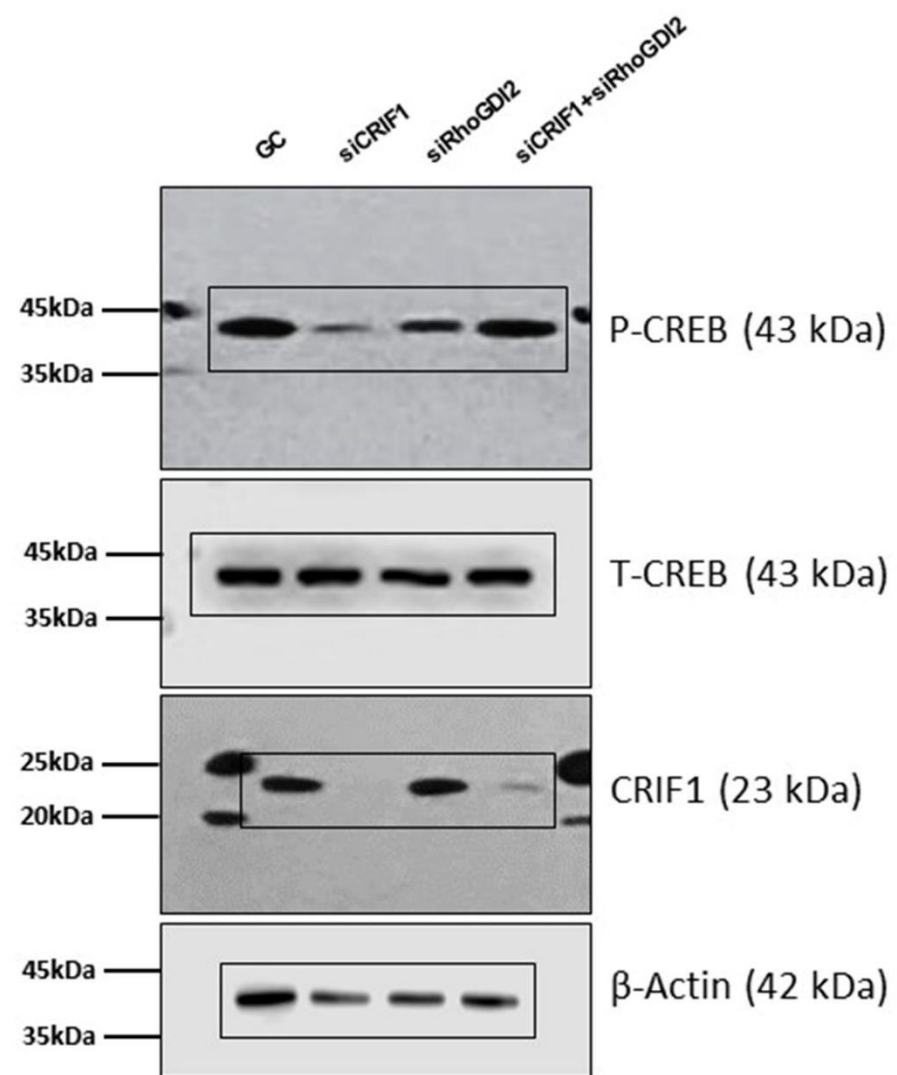

Fig 2A

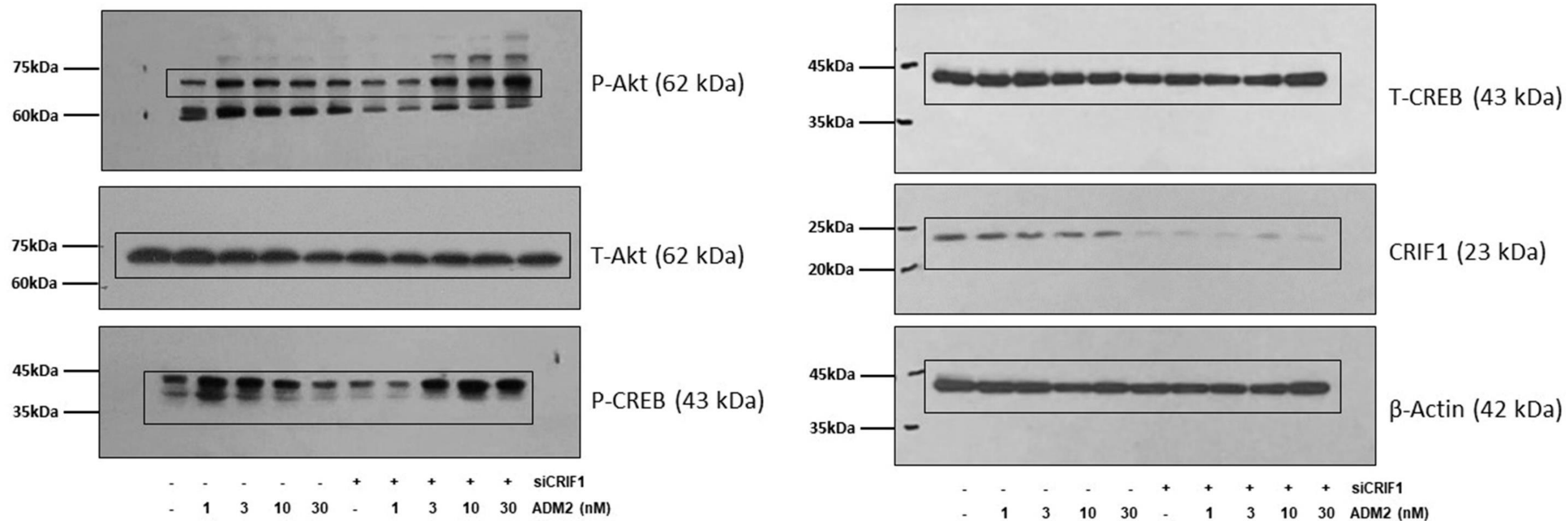

Fig 4D

Supplement: S1 Raw images — (PDF) [file pone.0256646.s001.pdf]
